# Optimized conditions for successful transfection of human endothelial cells with in vitro synthesized and modified mRNA for induction of protein expression

**DOI:** 10.1186/1754-1611-8-8

**Published:** 2014-03-03

**Authors:** Meltem Avci-Adali, Andreas Behring, Timea Keller, Stefanie Krajewski, Christian Schlensak, Hans Peter Wendel

**Affiliations:** 1 Department of Thoracic, Cardiac, and Vascular Surgery, University Hospital Tuebingen, Calwerstr. 7/1, 72076 Tuebingen, Germany

**Keywords:** mRNA synthesis, Transfection, Protein synthesis

## Abstract

**Background:**

The induction of protein synthesis by exogenous delivery of coding synthetic mRNA in desired cells is an auspicious strategy in the fields of basic cell biology, regenerative medicine, treatment of diseases, and reprogramming of cells. Here, we produced modified messenger RNA (mRNA) with reduced immune activation potential and increased stability and performed transfection experiments with different cells, HEK293 cells, BJ fibroblasts, and endothelial cells (ECs).

**Results:**

The mRNA induced protein expression in cells was analyzed after transfection with different mRNA amounts and transfection reagents using flow cytometry. Different cell types showed different degrees of eGFP expression. HEK293 cells exhibited the highest eGFP expression compared to the BJ fibroblasts and ECs. However, the mRNA induced eGFP expression was detected in all cell types until 3 days after transfection. Already, the use of 0.5 μg of the synthesized mRNA led to the significant expression of eGFP in ECs. From all analyzed ECs approximately 87% were eGFP positive, which showed a high transfection efficiency.

**Conclusions:**

The synthesis of stabilized mRNA and the high transfection efficiency will enable the mRNA engineering of ECs as well as other somatic cells. The delivery of synthetic exogenous mRNA into cells allows the transient expression of desired proteins, which would be normally not expressed by the cells.

## Background

**Figure 1:**
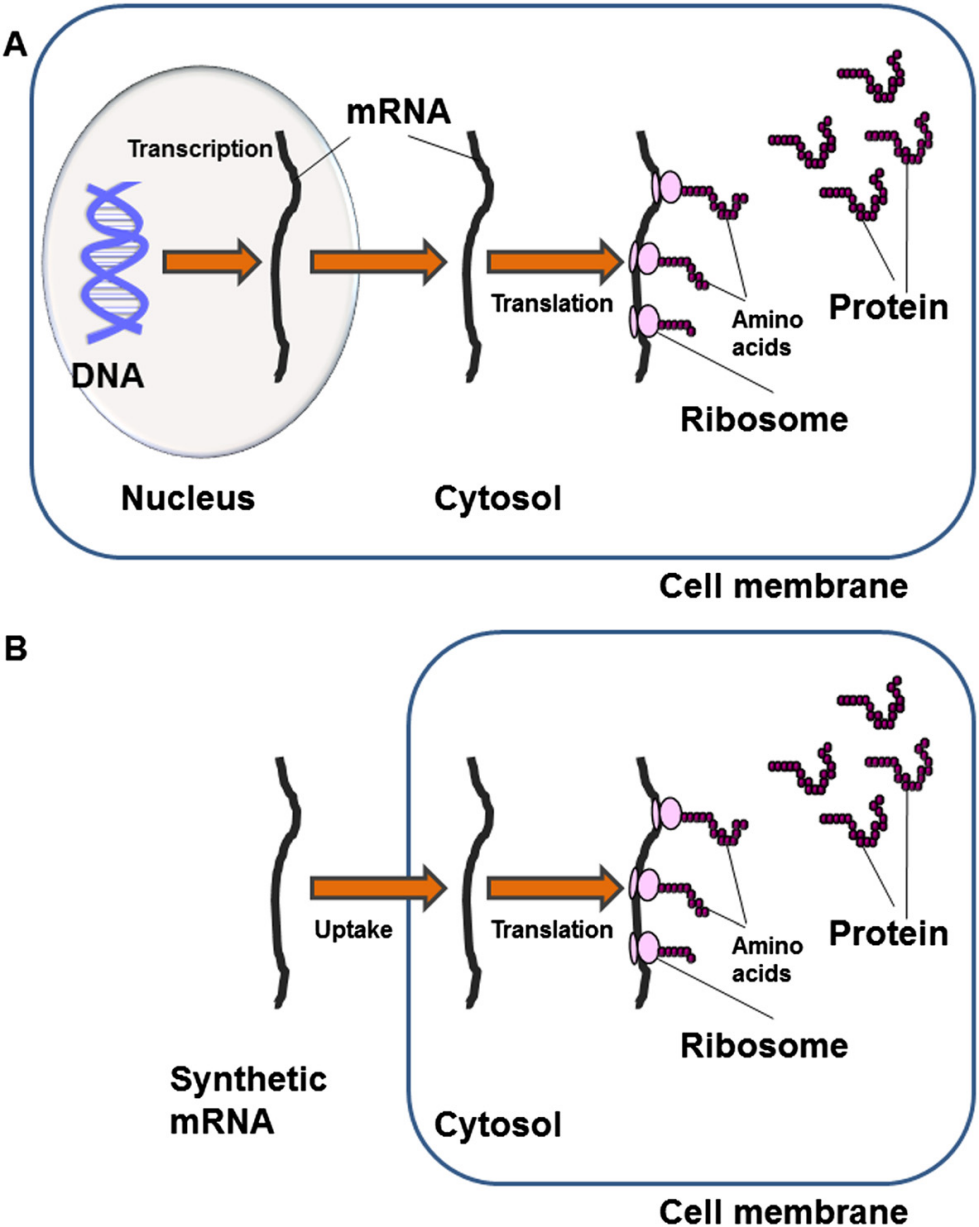
**Synthesis of proteins in the cells. A)** Natural pathway. Genetic code of the protein is transcribed into mRNA in the nucleus and transported to the cytosol. The mRNA is then translated by ribosomes into proteins. **B)** Synthetic pathway. Synthetically produced mRNA is transfected into the cells. The mRNA is then translated by ribosomes into proteins.

Proper functioning of cells, differentiation and production of signaling molecules are controlled via translation of messenger RNA (mRNA) into proteins by ribosomes (Figure 1). Thus, hereditary or acquired genetic disorders followed by defects of protein expression can lead to serious diseases. In the last years, the administration of mRNA as a therapeutic agent has gained an enormous potential in the fields of disease treatment [1], regenerative medicine [2-4], and vaccination [5]. Hitherto, clinical applications of conventional mRNAs were limited due to the low stability and the strong immunogenicity. However, pioneering work from Kariko and colleagues demonstrated that the replacement of cytidine and uridine by 5-methylcytidine and pseudouridine dramatically abrogate immune activation [6,7]. They also found that pseudouridine modified mRNAs have a higher translational capacity than unmodified mRNAs and increased biological stability [8,9].

The ability to produce modified mRNAs allows the protection of naturally labile mRNA in biological fluids and greatly minimizes innate immune responses. Kormann et al. demonstrated that a combination of nucleotide modifications prevents the interaction of synthesized mRNA with TLR3, TLR7, TLR8, and RIG-1 [1]. Thus, the use of modified mRNA provides the possibility for controlled and temporary delivery of desired transcripts in vivo or in vitro to induce protein expression. Furthermore, in comparison to the viral gene therapy vectors [10], the risk of oncogenesis [11] is prevented due to lack of integration into the host cell genome. Thus, the therapy with modified synthetic mRNA will get better clinical acceptance in the future.

Here, we synthesized modified eGFP mRNA and transfected different cell types, endothelial cells (ECs), BJ fibroblasts as well as HEK293 cells, with this mRNA. Transfection efficiency and mRNA induced eGFP expression in cells was compared after the transfection with different amounts of mRNA and different transfection reagents. Furthermore, the protein expression in transfected cells was measured up to 3 days after transfection.

## Materials and methods

### Production of modified mRNA

Coding DNA sequence (CDS) with known flanking sequences was amplified by PCR using specific primers. PCR product was then purified and the quality of the generated DNA was determined. Using the in vitro transcription (IVT) process, mRNA was generated from the DNA product. Subsequently, the product was purified and treated with phosphatase to remove 5′-triphosphates. After the additional purification and quality control of generated mRNA, transfection experiments were performed.

### Consent

ECs were isolated from residual saphenous vein biopsies of patients who had undergone coronary artery bypass graft surgery. All patients gave informed consent to participate and for the publication of this report. The study was approved by the Ethical Committee of the Faculty of Medicine of the University of Tuebingen.

#### Amplification of plasmid inserts and adding of polyT-tail by polymerase chain reaction (PCR)

To obtain, the DNA template for the IVT, pcDNA 3.3_eGFP Plasmid (Addgene, cat. no. 26822) containing CDS of interest, here eGFP, was amplified using PCR. The forward primer 5′-TTGGACCCTCGTACAGAAGCTAATACG-3′ and reverse primer 5′- T_120_-CTTCCTACTCAGGCTTTATTCAAAGACCA-3′ for the insert amplification were purchased from ELLA Biotech (Martinsried, Germany) in HPLC grade. Thereby, a poly T-tail of 120 thymidines (T) was added to the insert by using a reverse primer with a T_120_ extension. Thus, after IVT, the generated mRNAs obtained a poly A-tail with a defined length. PCR reactions of 100 μl were performed using HotStar HiFidelity Polymerase Kit (Qiagen, Hilden, Germany) and contained 0.7 μM of each forward and reverse primer, 1× Q-solution, 1× HotStar HiFidelity PCR buffer, 50 ng plasmid DNA, 2.5 U HotStar HiFidelity DNA polymerase. PCR was run using the following cycling protocol: initial activation step at 95°C for 5 min, followed by 25 cycles of denaturation at 95°C for 45 s, annealing at 55°C for 1 min, extension at 72°C for 1 min and final extension at 72°C for 10 min. PCR reaction was purified using QIAquick PCR Purification Kit (Qiagen, Hilden, Germany) according manufacturer’s instructions and the DNA was eluted using 20 μl nuclease-free water. The quality and purity of the DNA was assessed by 1% agarose gel electrophoresis.

#### In vitro transcription (IVT)

After the PCR, the genetic information is in vitro transcribed from DNA to mRNA using MEGAscript® T7 Kit (Life Technologies, Darmstadt, Germany). The generated mRNA transcript is then used to induce protein expression in cells. At first, 23 μl NTP/cap analog mixture containing 7.5 mM ATP, 1.875 mM GTP (both from MEGAscript® T7 Kit), 7.5 mM Me-CTP, 7.5 mM Pseudo-UTP (both from TriLink BioTechnologies, San Diego, USA), and 2.5 mM 3′-O-Me-m^7^G(5′)ppp(5′)G RNA cap structure analog (New England Biolabs, Frankfurt am Main, Germany) was prepared and mixed thoroughly. The IVT reaction mixture of 40 μl was then assembled by adding 40 U RiboLock RNase inhibitor (Thermo Scientific, Waltham, USA), 1 μg PCR product, 1× reaction buffer and 1× T7 RNA polymerase enzyme mix. The IVT reaction mixture was incubated at 37°C for 3 h in a thermomixer. To remove the template DNA, 1 μl TURBO DNase (from MEGAscript® T7 Kit) was added to the IVT reaction mixture and incubated for 15 min at 37°C. Then, the reaction mixture was purified using RNeasy Mini Kit (Qiagen, Hilden, Germany) according to manufacturer’s instructions. The modified mRNA was eluted from the spin column membrane twice with 40 μl nuclease-free water.

#### Treatment of purified mRNA with Antarctic phosphatase

The generated mRNA is treated with Antarctic phosphatase (New England Biolabs, Frankfurt am Main, Germany) to remove 5′ triphosphates, which can be recognized by RIG-1, and lead to the immune activation. Furthermore, the phosphatase treatment prevents the recircularization in a self-ligation reaction. For this purpose, 9 μl of 10× Antarctic phosphatase reaction buffer was added to 79 μl of purified mRNA solution. Subsequently, 2 μl of Antarctic phosphatase (5 U/μl) was added to the reaction mixture and incubated at 37°C for 30 min. The treated mRNA was purified using RNeasy Mini Kit (Qiagen, Hilden, Germany) according to manufacturer´s instructions. The modified mRNA was eluted from the spin column membrane twice with 50 μl nuclease-free water. The concentration was measured using ScanDrop spectrophotometer (Analytic Jena, Jena, Germany). The concentration of mRNA was adjusted to 100 ng/μl by adding nuclease-free water. The quality and purity of synthesized modified mRNA was determined by 1% agarose gel electrophoresis. The modified mRNA was aliquoted and stored at -80°C and used for transfections.

### Cultivation of cells

HEK293 cells were cultivated in DMEM with high glucose (PAA Laboratories, Cölbe, Germany) supplemented with 10% FBS (Life Technologies, Darmstadt, Germany), 2 mM L-glutamine (PAA Laboratories), and 1% penicillin/streptomycin (PAA Laboratories). BJ human foreskin fibroblasts (Stemgent, Cambridge, USA) were cultivated in DMEM with high glucose containing 10% FBS, 2 mM L-glutamine, 1% penicillin/streptomycin, and 30 mM HEPES (Life Technologies, Darmstadt, Germany). Human endothelial cells (ECs) were isolated as described previously [12] and cultivated in flasks precoated with 0.1% gelatin in Vasculife® EnGS EC culture medium (CellSystems, Troisdorf, Germany) containing VascuLife EnGS LifeFactors Kit, 50 mg/ml gentamicin, and 0.05 mg/ml amphotericin B (PAA Laboratories). Cells were kept at 37°C with 5% CO_2_ and media was changed every 3 days. Cells were passaged using trypsin/EDTA (0.04%/0.03%, PromoCell, Heidelberg, Germany). BJ fibroblasts at passage 7-9, endothelial cells at passage 3-5 were used for all experiments.

#### Preparing of cells for mRNA transfection

For performing of transfection experiments, 1.5 × 10^5^ cells were plated per well of 12-well plate. The cells were incubated overnight at 37°C in a cell incubator. Next day, transfection experiments were performed.

#### Detachment of cells and performing of flow cytometry

Using trypsin/EDTA (0.04%/0.03%), cells were detached, washed, and fixed in 1× CellFix solution (BD Biosciences, Heidelberg, Germany). A total of 10,000 cells was analyzed by FACScan flow cytometer (Becton Dickinson, Heidelberg, Germany). Cells expressing eGFP were detected using the FL1 (green) channel and measuring the fluorescence at 509 nm. FACS data were displayed in histograms. For the evaluation, a marker M1 was set, which contained maximum of approximately 1.5% of control cells (cells treated with transfection medium without mRNA). Using histogram statistics of the CellQuest Pro Software (Becton Dickinson), the percentage of eGFP positive cells was determined to assess the transfection efficiency. Geo Mean (geometric mean) values show the strength of the fluorescence intensity. Higher Geo Mean values reflect stronger fluorescent intensity, indicating a higher production of eGFP.

### Performing of transfections with different transfection reagents

Expression of eGFP was verified after performing of transfection with 1.5 μg mRNA, using TransIT mRNA transfection reagent (Mirus Bio LLC, Madison, USA), Lipofectamine® 2000 (Life Technologies, Darmstadt, Germany), or Stemfect RNA transfection reagent (Stemgent, Cambridge, USA). From each transfection reagent 2 μl was used for transfections. The cells were incubated for 4 h with transfection complexes, subsequently the transfection mixture was replaced by cell culture medium. The expression of eGFP was measured 24 h after transfection.

To perform the transfection with Trans-IT-mRNA transfection reagent, 1.5 μg mRNA, 2 μl TransIT-mRNA transfection reagent and 2 μl boost reagent were added to 100 μl Opti-MEM I reduced serum media (Life Technologies, Darmstadt, Germany) and incubated for 5 min at room temperature. Afterwards, transfection complexes were added for 4 h to 1 ml cell medium in 12 well plates.

Two tubes containing 25 μl Stemfect transfection buffer were prepared for assembling of the Stemfect RNA transfection mixture. In the first tube, 2 μl of Stemfect RNA transfection reagent was added. In the second tube, 1.5 μg of mRNA was added. Then the diluted transfection reagent solution was pipetted to the diluted mRNA solution, mixed, and incubated for 15 minutes at room temperature. The mRNA transfection complex was added for 4 h to 1 ml cell medium in 12 well plates.

Using the transfection reagent Lipofectamine 2000, two different transfections were performed. Lipofectamine 2000/mRNA complexes were added to the cell culture media or given only in Opti-MEM I reduced serum media to the cells. For transfection of cells only with Opti-MEM I reduced serum media, the Lipofectamine 2000 transfection mixtures were prepared by addition of 2 μl Lipofectamine 2000 and 1.5 μg mRNA to 500 μl Opti-MEM I reduced serum media and incubation for 20 min at room temperature. Cell culture medium was aspirated, cell layer was washed with 1 ml DPBS and transfection mixture was given on adherent growing cells for 4 h at 37°C. This transfection method is referred to as Lipofectamine 2000_optimized. For transfection of cells in cell culture medium, 2 μl Lipofectamine 2000 and 1.5 μg mRNA were added to 100 μl Opti-MEM I reduced serum media and incubated for 5 min at room temperature. Then, the mixture was added to 1 ml cell culture medium per well of a 12-well plate for 4 h at 37°C.

### Transfection of cells with different Lipofectamine 2000 amounts

To determine the required amount of Lipofectamine 2000 for forming of lipoplexes, different amounts of Lipofectamine 2000, 1, 2, 4, 6 μl, were used to transfect the cells. For transfection of one well of 12-well plate, 500 μl Opti-MEM I reduced serum media was prepared containing 2.5 μg of eGFP mRNA and respective amount of Lipofectamine 2000. The components were gently mixed by pipetting. The transfection mixture was then incubated at room temperature for 20 min to generate lipoplexes for transfection. Cells were washed with 500 μl DPBS/well, the transfection mixture was pipetted into the well. After 4 h incubation at 37°C and 5% CO_2_, the transfection mixture was replaced by 1 ml complete cell culture medium. Cells were cultivated for 24 h in the cell incubator and analyzed using flow cytometry.

### Transfection of cells with different mRNA amounts

To determine the required amount of mRNA for induction of protein expression, different amounts of eGFP mRNA, 0, 0.5, 1, 1.5, 2, 2.5 μg, were used to perform the transfection of cells. For transfection of one well of 12-well plate, 500 μl Opti-MEM I reduced serum media with respective amount of mRNA and 2 μl of Lipofectamine 2000 was prepared. The components were gently mixed and incubated 20 min at room temperature. Cells were washed with 500 μl DPBS/well and the transfection mixture was added. Cells were incubated for 4 h at 37°C and 5% CO_2_. Afterwards, the transfection mixture was aspirated and 1 ml complete cell culture medium was added to the cells. Cells were incubated for 24 h in the cell incubator. Using flow cytometry, the eGFP expression in the cells was verified.

### Determination of eGFP expression in mRNA transfected cells over time

To evaluate the duration of protein expression in cells, eGFP expression was determined 1, 2, and 3 days after mRNA transfection. Transfection complexes were generated in 500 μl Opti-MEM I reduced serum media by adding of 2.5 μg eGFP mRNA and 2 μl Lipofectamine 2000. The components were incubated at room temperature for 20 min to form lipoplexes. Cells were washed with 500 μl DPBS/well. Subsequently, cells were incubated for 4 h at 37°C and 5% CO_2_ with the transfection mixture. Then, the mixture was aspirated and 1 ml complete cell culture medium was added to the cells. Cells were cultivated for 1, 2, and 3 days in the cell incubator and analyzed using flow cytometry.

### Fluorescence microscopic analysis of eGFP transfected cells

Using fluorescence microscopy, the expression of eGFP in HEK293 cells, BJ fibroblasts, and ECs was examined. The cells were transfected with 1.5 μg eGFP mRNA using 2 μl Lipofectamine 2000. Fluorescence micrographs were taken 24 h post-transfection.

### Statistical analysis

Data are shown as means ± standard deviation (SD). One-way analysis of variance (ANOVA) for repeated measures followed by the Bonferroni’s multiple comparison test was performed to compare the means. All statistical analyses were performed double-tailed using GraphPad Prism version 5.01. Differences of p < 0.05 were considered significant.

## Results

### Performing of mRNA transfection with different transfection reagents

**Figure 2:**
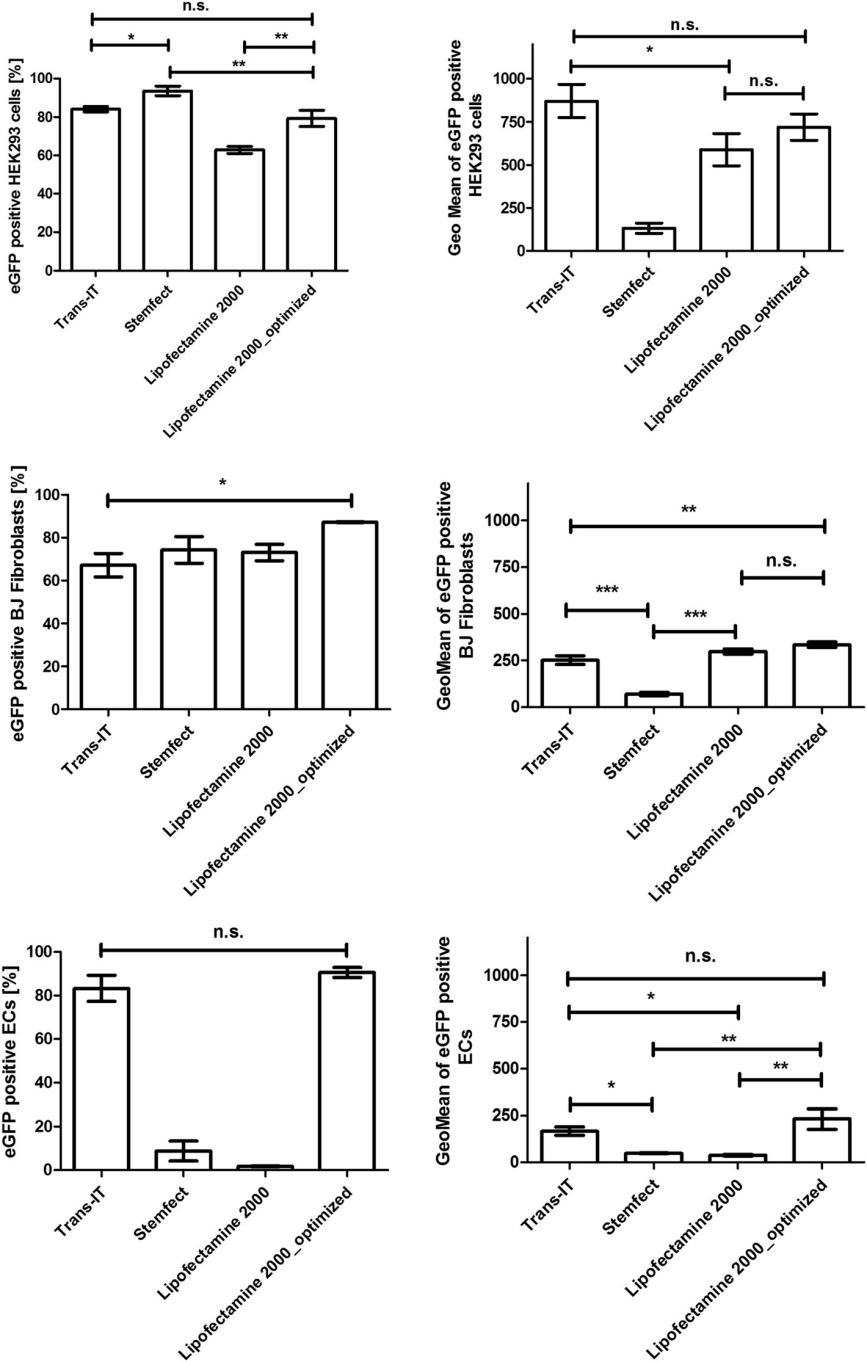
Transfection of HEK293 cells, BJ fibroblasts, and ECs with 1.5 μg eGFP mRNA using different transfection reagents and modifications.

HEK293 cells, BJ fibroblasts, and ECs were transfected with 1.5 μg mRNA using TransIT mRNA transfection reagent, Lipofectamine 2000, or Stemfect RNA transfection reagent (Figure 2).

Transfection of HEK293 cells using Stemfect RNA transfection reagent led to the highest transfection efficiency, approximately 94% of cells expressed eGFP. However, the eGFP expression in cells measured by fluorescence intensity was lowest compared to the other transfection reagents. The transfection efficiency using TransIT mRNA transfection reagent and Lipofectamine 2000_optimized was similar with approximately 80% eGFP expressing cells. The fluorescence intensity in these cells was also not significantly different.

Using Stemfect RNA transfection reagent, about 74% of the analyzed BJ fibroblasts were transfected with mRNA. However, like in HEK293 cells, the protein expression in the BJ fibroblasts was lowest after the transfection with Stemfect RNA transfection reagent compared to other transfection reagents. Lipofectamine 2000_optimized resulted in best transfection efficiency with approximately 87% eGFP positive cells.

Very low amounts of ECs could be transfected using Stemfect RNA transfection reagent and Lipofectamine 2000/mRNA complexes in cell culture medium. The transfection using Trans-IT mRNA transfection reagent or Lipofectamine 2000_optimized resulted in high transfection efficiency. The eGFP expression in cells was not significantly different after transfection with both transfection reagents.

### Transfection of cells with different Lipofectamine 2000 amounts

**Figure 3:**
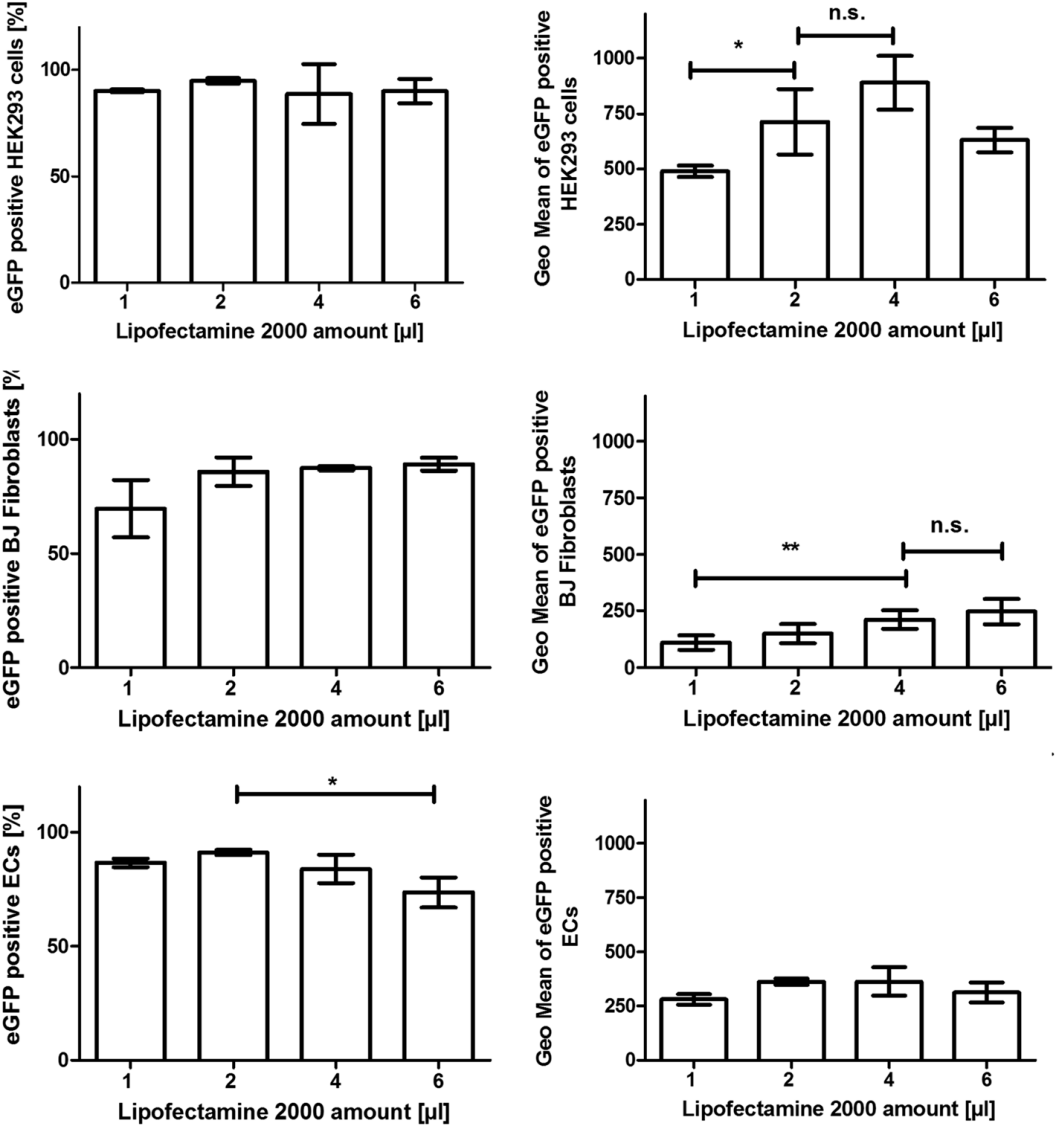
Transfection of HEK293 cells, BJ fibroblasts, and ECs with 2.5 μg eGFP mRNA using different amounts of Lipofectamine 2000.

HEK293 cells, BJ fibroblasts, and ECs were transfected with 2.5 μg eGFP mRNA using 1, 2, 4, and 6 μl of Lipofectamine 2000 for generation of transfection complexes (Figure 3).

The percentage of eGFP expressing HEK293 cells was not significantly different between the transfections with different Lipofectamine 2000 amounts. Approximately 91% of the cells were eGFP positive. However, the use of 2 μl Lipofectamine 2000 resulted in significantly higher fluorescence intensity (Geo Mean) in HEK293 cells than after the transfection with 1 μl Lipofectamine 2000. The use of 4 μl Lipofectamine 2000 did not lead to significantly higher fluorescence intensity than the transfection with 2 μl. After the transfection with 2 μl Lipofectamine 2000, the Geo Mean was approximately 710.

The transfection of BJ fibroblasts with different Lipofectamine 2000 amounts did not result in significantly different percentage of eGFP positive cells. Approximately 83% of the cells were positive for eGFP expression. The use of 4 μl Lipofectamine 2000 showed significantly higher Geo Mean compared to the use of 1 μl Lipofectamine. Using 4 μl Lipofectamine 2000, a Geo Mean of 210 was detected.

The performance of EC transfection with 6 μl Lipofectamine 2000 led to decreased percentage of eGFP positive cells compared to the transfection with 2 μl Lipofectamine 2000. The transfection with 1, 2, and 4 μl Lipofectamine 2000 resulted in approximately 87% eGFP positive cells. The use of these different Lipofectamine 2000 amounts for transfection showed no significant differences. Using 2 μl Lipofectamine 2000, a Geo Mean of 362 was detected.

### Transfection of cells with different mRNA amounts

**Figure 4:**
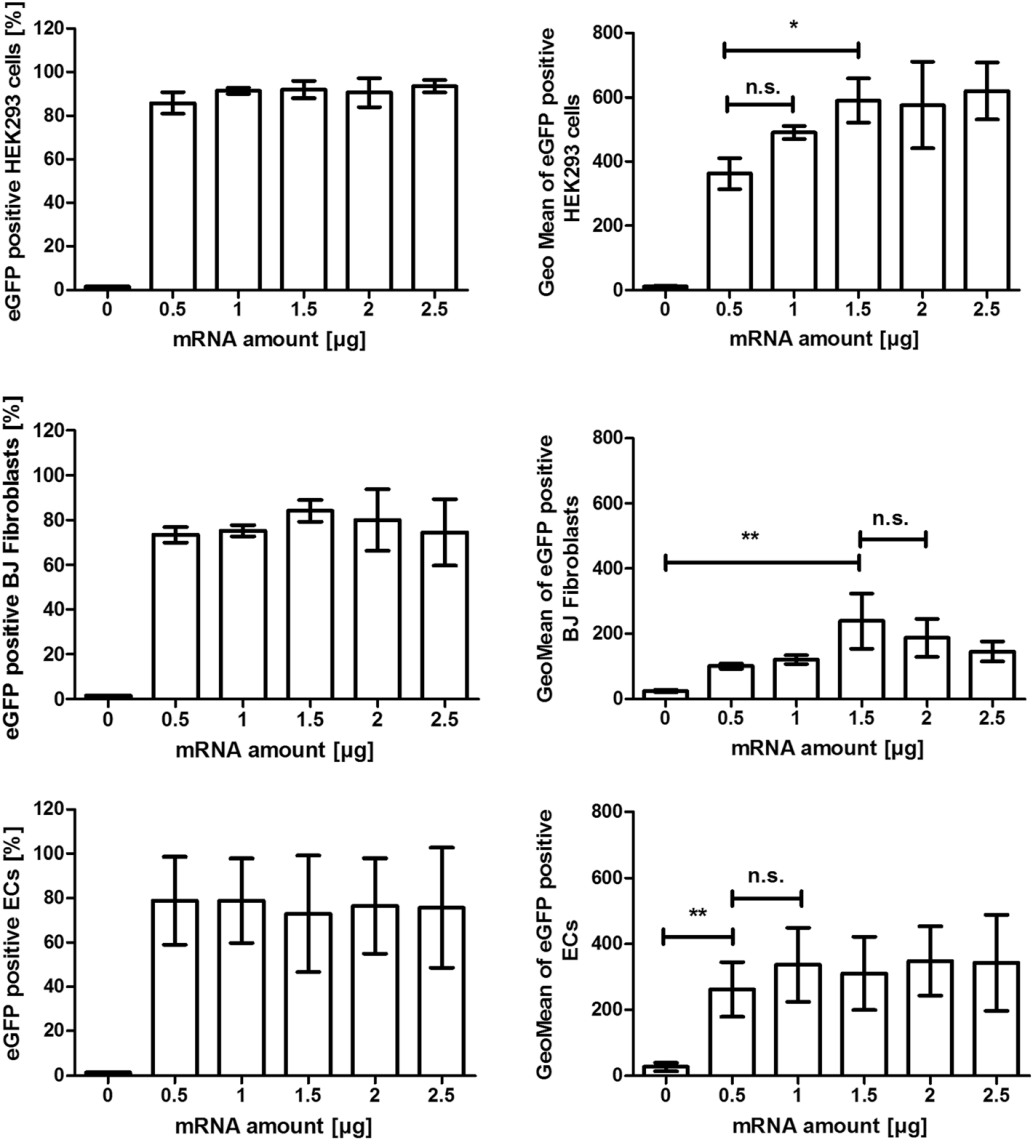
Transfection of HEK293 cells, BJ fibroblasts, and ECs with 2 μl Lipofectamine 2000 and different amounts of eGFP mRNA.

The mRNA transfection complexes were generated by using 2 μl Lipofectamine 2000 and different amounts of eGFP mRNA, 0, 0.5, 1, 1.5, 2, and 2.5 μg. After the transfection of HEK293 cells, BJ fibroblasts, and ECs, the percentage of eGFP positive cells and the Geo Mean was determined using flow cytometry (Figure 4).

Already the transfection of HEK293 cells with 0.5 μg mRNA resulted in detection of 86% eGFP positive cells. The increasing of the mRNA amount did not significantly increase the percentage of transfected cells. But the use of 1.5 μg mRNA led to higher eGFP expression in cells than the use of 0.5 μg mRNA (Geo Mean of 590 versus 363). However, the transfection of cells with more than 1.5 μg mRNA did not increase the eGFP expression in the cells.

The transfection of BJ fibroblasts with 0.5 μg mRNA led to the transfection of 75% of the cells. The increasing of the mRNA amount did not result in significantly higher transfection efficiency. The transfection of 1.5 μg mRNA led to significantly higher fluorescence intensity than the negative cells. The Geo Mean was 239.

The use of only 0.5 μg mRNA led to the transfection of 79% of all ECs. The transfection efficiency was not enhanced by increasing the mRNA amount. Already 0.5 μg mRNA led to significantly higher expression of eGFP in ECs than in the negative cells. The detected Geo Mean was 262. The use of higher amounts of mRNA did not lead to increased eGFP synthesis.

### Determination of eGFP expression in mRNA transfected cells over time

**Figure 5:**
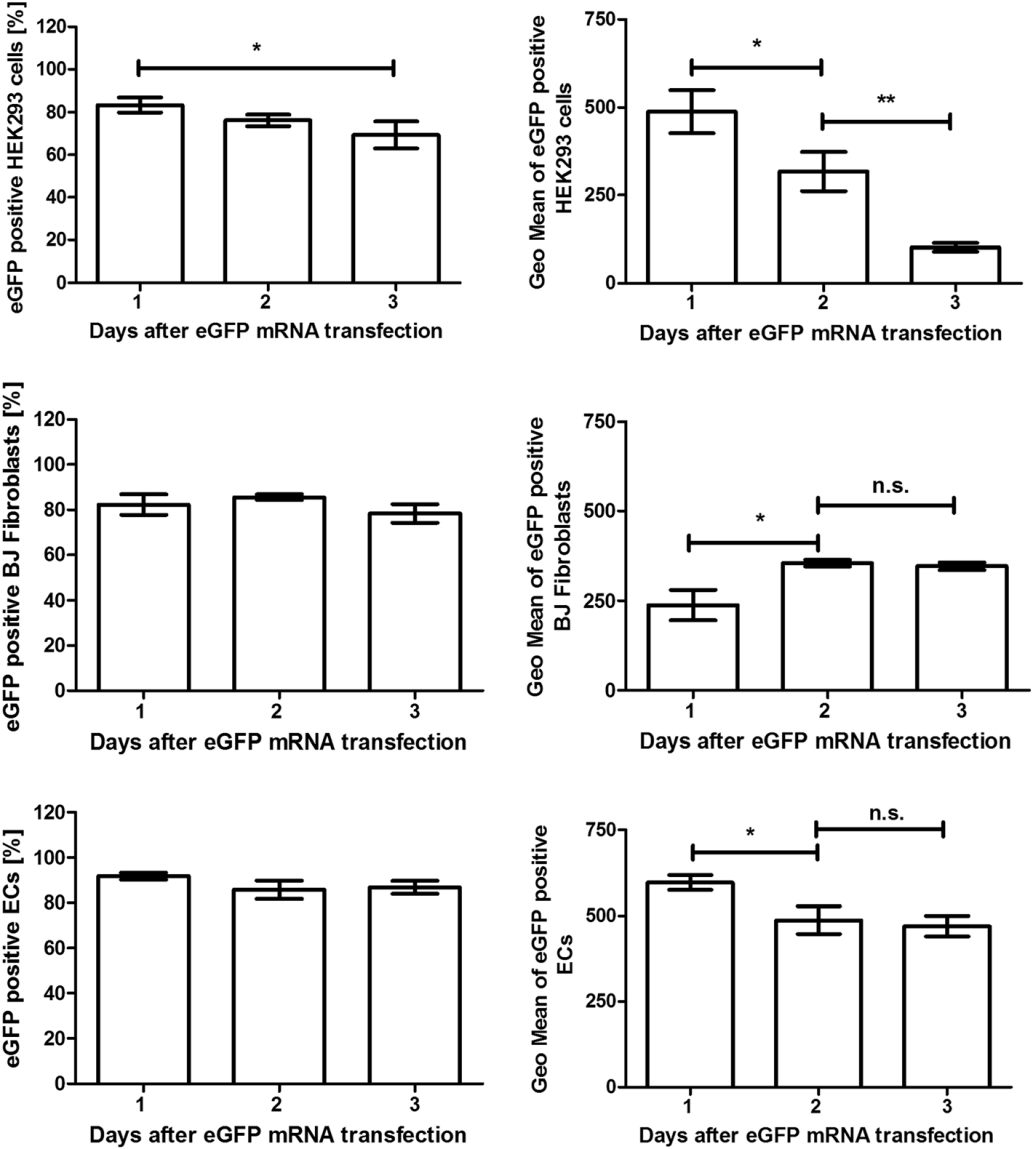
Transfection of HEK293 cells, BJ fibroblasts, and ECs with eGFP mRNA and determination of eGFP expression for 3 days.

The duration of synthetic mRNA induced protein expression in HEK293 cells, BJ fibroblasts, and ECs was determined after the transfection of cells with 2.5 μg mRNA using 2 μl Lipofectamine 2000. The eGFP expression in cells was determined 1, 2, and 3 days after transfection using flow cytometry (Figure 5).

Flow cytometry analysis of eGFP mRNA transfected HEK293 cells showed a significant decrease of the portion of eGFP producing cells after 3 days. However, after 3 days, 69% of analyzed cells were still positive for eGFP expression. Over time, a continuous decrease of eGFP expression in the cells was detected. After 3 days, the detected Geo Mean was approximately 4 times less than 1 day after transfection.

The percentage of eGFP positive BJ fibroblasts did not significantly change over time. But the eGFP expression in the cells was 2 days post-transfection higher than after 1 day. The fluorescence intensity of the cells did not change after 3 days.

Approximately 88% of ECs were positive for eGFP expression and the amount of eGFP positive cells did not change during 3 days. The eGFP expression peak was measured 1 day post-transfection. A significant decrease of Geo Mean was detected 2 days after transfection. However, the detected fluorescence intensity did not change in the following day.

### Fluorescence microscopic analysis of eGFP transfected cells

**Figure 6:**
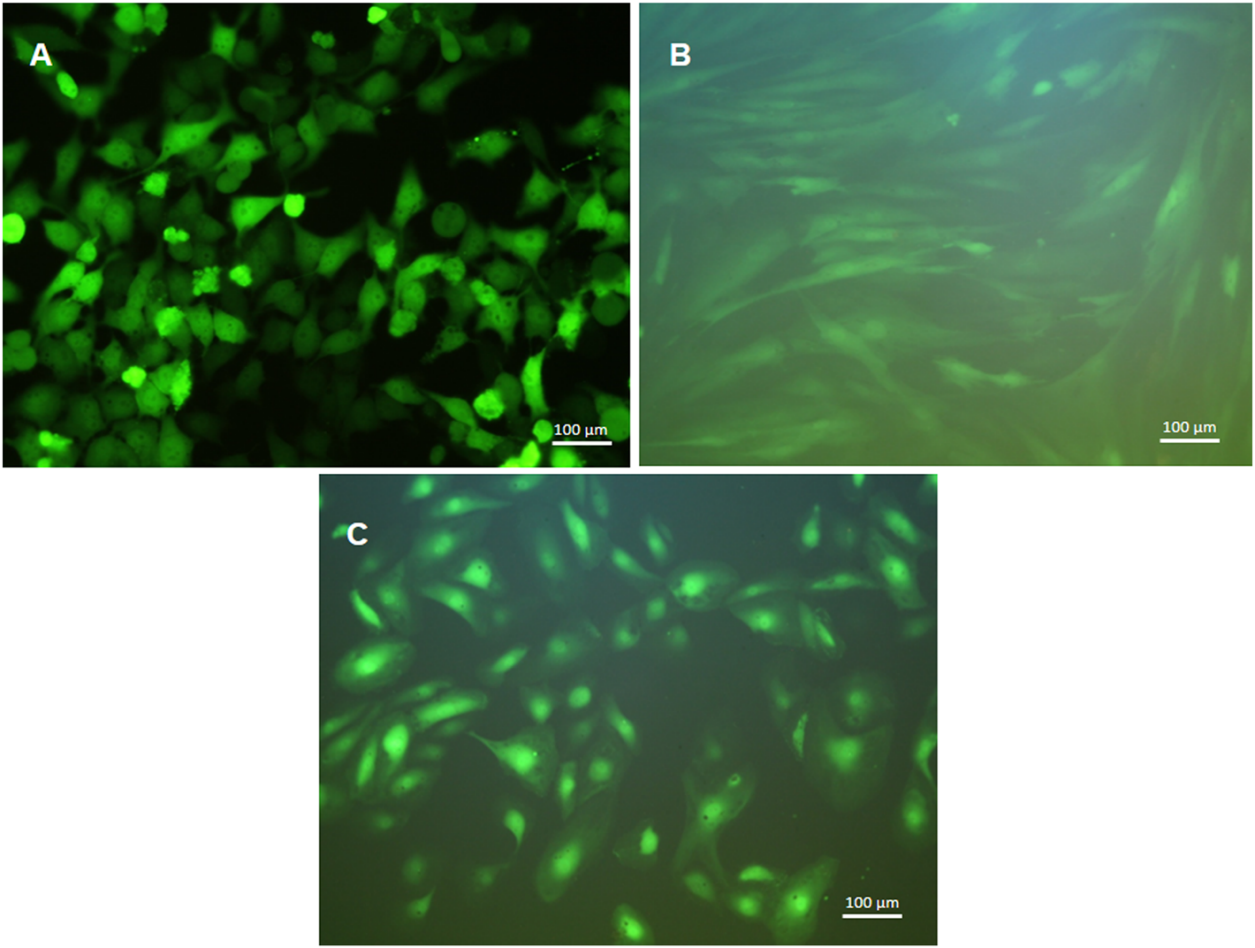
Fluorescence microscopic analyses of A) HEK293 cells, B) BJ fibroblasts, and C) ECs 24 h after eGFP mRNA transfection.

HEK293 cells, BJ fibroblasts, and ECs were transfected with 1.5 μg eGFP mRNA using 2 μl Lipofectamine 2000. After 24 h, the eGFP expression in cells was investigated by fluorescence microscopy (Figure 6). The analyses demonstrated strongly fluorescent HEK293 cells. The fluorescence intensity of BJ fibroblasts was weaker than of the ECs.

## Discussion

In recent years, the induction of protein expression in cells by delivery of synthetic modified mRNA gained in importance. Thereby, cells can be triggered to synthesize proteins for treatment of diseases caused by inability of cells to produce a key protein. For example genetic disorders can be corrected by introduction of mRNA that codes for the missing or defective protein [13]. Furthermore, mRNA therapeutics can also be used as vaccines to synthetize protein antigens for activation of the host immune system to effectively eliminate tumor cells or prevent infections [5,14]. Moreover, mRNA was also successfully used to reprogram finally differentiated somatic cells into pluripotent stem cells by the expression of reprogramming factors [3]. In a recent study, MSCs were mRNA engineered to synthetize increased amounts of proteins for enhanced regenerative potential [15].

Here, modified eGFP mRNA was synthetized and the mRNA transfection behavior of different cell types, primary cells such as ECs, BJ fibroblasts, and the HEK293 cell line, was analyzed. The determined optimal conditions can be used to achieve maximal transfection efficiency and expression of desired proteins especially in ECs. But above all, we demonstrated the successful transfection of ECs with synthetic mRNA, which resulted in production of functional protein.

Firstly, the appropriate transfection reagent and the transfection conditions were determined. The transfection of ECs with Stemfect RNA transfection reagent and Lipofectamine 2000 in cell culture medium resulted in very low levels of transfection. In contrast, transfection of cells with Lipofectamine 2000/mRNA complexes in Opti-MEM I reduced serum media or the use of TransIT mRNA transfection reagent/mRNA complexes in cell culture medium led to a high transfection efficiency between 80 and 90%. The measurements also demonstrated that HEK293 cells can be successfully transfected with high efficiency using TransIT mRNA transfection reagent or Lipofectamine 2000_optimized method. BJ fibroblasts showed highest transfection efficiency using Lipofectamine 2000_optimized method.

The use of transfection reagents in low amounts can lead to reduced transfection efficiency and on the other hand when they are used in high amounts, they can induce cell toxicity. Thus, the use of optimal mRNA to lipofectamine ratio is important to maintain cell viability and high transfection efficiency. The transfection of HEK293 cells, BJ fibroblasts, and ECs was performed with different amounts of Lipofectamine 2000. The measurements showed that the transfection of BJ fibroblasts can be performed using 4 μl Lipofectamine 2000, for HEK293 cells 2 μl was sufficient and the transfection of ECs can be performed with 1 or 2 μl Lipofectamine 2000. The use of 6 μl Lipofectamine 2000 for transfection of ECs resulted in decreased transfection efficiency, which indicates a toxic effect of Lipofectamine 2000 in higher concentrations. This shows that the Lipofectamine 2000 concentration should be optimized for each cell type.

After the mRNA transfection, HEK293 cells showed higher fluorescence intensities than ECs and BJ fibroblasts, which means that they produce higher amounts of eGFP. In comparison to ECs with a Geo Mean of 362 and BJ fibroblasts with a Geo Mean of 210, the Geo Mean of HEK293 cells was 710. This was also confirmed by fluorescence microscopic analyses. However, despite different fluorescence intensities, all cell types demonstrated a high transfectability.

The transfection of cells already with 0.5 μg mRNA and 2 μl Lipofectamine 2000 resulted in a high transfection efficiency, 86% of HEK293 cells, 75% of BJ fibroblasts, and 79% of ECs were eGFP positive. However, the increasing of mRNA amount from 0.5 μg to 1.5 μg led to significantly increased eGFP synthesis in HEK293 cells. The transfection of BJ fibroblasts with 1.5 μg mRNA was needed to detect significantly higher expression of eGFP in the cells compared to the control cells. In contrast, the transfection of ECs only with 0.5 μg eGFP mRNA was sufficient to induce significantly increased protein expression compared to the control cells. The expression did not change by increasing the mRNA amount to 2.5 μg.

Only after a single transfection with eGFP mRNA, all cell types expressed eGFP for 3 days. Thus, these results showed that by using synthetic modified mRNA the protein expression can be maintained over a few days in transfected cells. The eGFP expression reached the maximum on day 2 in BJ fibroblasts and showed no change on day 3. In ECs, the eGFP expression was highest 1 day post-transfection and decreased after 2 days. However, the detected fluorescence intensity after 3 days was still high with a Geo Mean of approximately 470. Each day, the eGFP expression continuously decreased in HEK293 cells. These cells are rapidly dividing cells with a doubling time of approximately 24 h. Thus, the rapid decrease of the fluorescence intensity compared to BJ fibroblasts and ECs could be caused by halving of the eGFP amount after each division.

## Conclusion

Here, we described the production of modified mRNA for exogenous delivery into cells to induce protein expression. The delivery of the generated mRNA led to the synthesis of eGFP in ECs, which was present during 3 days in the cells. However, the results also demonstrated that the transfection method, reagent, and the amount of mRNA and transfection reagent influence the successful mRNA delivery and thus the expression of the protein. Using the exogenous mRNA delivery, each cell type could be induced to temporarily produce the desired proteins. This method has great potential for therapeutic applications, but each application has to be first optimized individually.

## Competing interests

The authors declare that they have no competing interests.

## Authors’ contributions

Conceived and designed the experiments: MAA, HPW, CS. Performed the experiments: AB, TK. Contributed reagents/materials/analysis tools: HPW, CS. Wrote the paper: MAA, HPW. Analyzed and interpreted the data: MAA, AB, SK. All authors read and approved the final manuscript.
